# How to eliminate (and even reverse) egocentric bias in perspective taking

**DOI:** 10.1177/17470218251341289

**Published:** 2025-05-08

**Authors:** Steven Samuel, Geoff G. Cole, Madeline J Eacott, Rebecca Edwardson

**Affiliations:** 1 Department of Psychology, City St. George’s, University of London, School of Health & Medical Sciences, London, UK; 2 School of Psychology, University of Plymouth, Plymouth, UK; 3 Department of Psychology, University of Essex, Colchester, UK

**Keywords:** Perspective taking, level 2 perspective taking, egocentric bias, embodiment, theory of mind

## Abstract

The ability to be objective about the perspectives of others is often compromised by interference from our own knowledge, beliefs and perceptions – an egocentric bias. However, recent research in visual perspective taking has found that this bias is eliminated immediately following a trial in which an alternative visual perspective is taken, suggesting egocentricity is flexible and can be eliminated under certain conditions. We examined such flexibility in relation to manual action. In contrast to other domains of perspective taking where egocentricity is usually problematic, egocentricity in manual action, even in the context of perspective taking, is usually useful, enabling accurate goal-directed movements based on real spatial relationships between the self and the environment (rather than on an imagined perspective). Eliminating egocentricity in manual action would thus make a particularly strong case for the flexibility of perspective taking. In four experiments, we assessed whether this ‘useful’ egocentric bias is compromised by practice on visual perspective taking tasks. Results showed that practice disambiguating stimuli from other perspectives makes manual actions consistent with second-person perspectives as easy as actions based on the first-person perspective for an equivalent time period afterwards.

Adults often display egocentric bias when attempting to be objective about others’ perspectives. For example, we sometimes fail to recognise that others do not share our opinions (Mullen et al., 1985; Ross et al., 1977), expertise (Hinds, 1999), valuations of objects (Van Boven et al., 2000), tactile sensations (Silani et al., 2013), feelings of discomfort (Nordgren et al., 2011; O’Brien & Ellsworth, 2012) and even thirst (Van Boven & Loewenstein, 2003). In visual perspective taking (VPT), we tend to process another agent’s viewpoint more slowly than our own (Chiu et al., 2019), imagine others are referring to objects that only we can see (Apperly et al., 2010; Keysar et al., 2003) and experience more interference from our own perspective than from others’ (Keysar et al., 2003; Samuel et al., 2020; Samuel, Eacott, et al., 2022; Wardlow Lane & Ferreira, 2008). Egocentric bias can make it difficult to think objectively (Risen & Critcher, 2011) and can give rise to misconceptions that can lead to social conflict (Van Boven et al., 2000).

What, therefore, might serve to reduce, eliminate, or even reverse egocentric bias? One candidate is that bias might decay with practice assuming different viewpoints. There is some evidence in favour of such a possibility. For example, Samuel, Roehr-Brackin, et al. (2019) gave adults a computerised version of a Director Task (Apperly et al., 2010; Keysar et al., 2003) in which participants interpreted instructions from either an avatar who shared their visual perspective of an array of objects (self-perspective trials) or an avatar on the other side of the array whose view of the objects was limited by occlusions (other-perspective trials). Participants needed to click on the object that each avatar named, bearing in mind that on other-perspective trials the avatar would only refer to what they could see. This meant that an accurate interpretation of an instruction from this avatar to select the ‘top cup’ sometimes required the participant to click on what was from their own perspective the *middle* cup if the actual top cup was occluded. Consistent with an egocentric bias in perspective taking, participants were faster to respond to instructions from the avatar who shared their perspective than the one with the restricted view. However, speed on self-perspective trials was similar to those of other-perspective trials when a self-perspective trial immediately followed an other-perspective trial. The authors argued that egocentricity is flexible and prone to disappear immediately following a perspective taking action (see also Brown-Schmidt & Hanna, 2011; Yoon & Brown-Schmidt, 2014).

The flexibility demonstrated in this study was short-lived, as results still pointed to an egocentric bias across the task as a whole. Stronger evidence for flexibility would come from a more lasting effect of taking other perspectives. It would also be useful to investigate whether this flexibility extends to another domain of performance. In the present studies, we examined the effect of perspective taking on manual action. Manual action represents a particularly interesting prism through which to look at egocentricity because there exist a priori reasons to suggest that it might be both harder *and* easier to eliminate egocentricity in this domain.

It might be *harder* to eliminate egocentricity in manual action because while it is often useful to understand others’ perspectives it is very rarely useful for us to make manual actions consistent with other points of view. For example, if I ask you to disambiguate a target from distractors from my own perspective, but then make a motor movement consistent with its spatial location from your perspective (e.g. touch it – see green line in Figure 1), activating the manual action that *I* would be required to make to reach for it (red line) could slow your response, or lead you to make an error. Therefore, in manual actions egocentricity is rarely a nuisance but instead a necessary component of successful goal-directed physical movement in the real world. Indeed, we could just as easily term this an egocentric *advantage*. As such, it should be difficult for practice with other perspectives to impact such a fundamental form of egocentricity.

**Figure 1.**
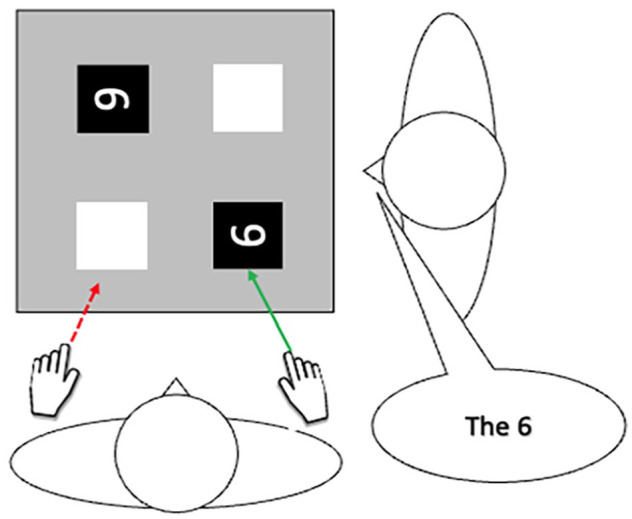
If asked to touch the 6 by the agent on the right, activating a representation of that agent’s motor movement (red line) could lead to competition that slows performance.

However, we could also predict that it is easier to eliminate egocentric bias in manual action because perspective taking can integrate our motor representations with the desired perspective, a process usually termed *embodied perspective taking* (Kessler & Rutherford, 2010; Kessler & Thomson, 2010; Samuel, Legg, et al., 2019; Surtees et al., 2013). Embodied perspective taking implies that in order to understand what is on another person’s left or right or how an object appears to that person, we perform a virtual alignment of our bodies with that of the target agent or perspective such that what is on their left is now represented on our own left; the left side of an object to the target agent is now the left side of the object for us, and so on. Support for embodied perspective taking comes from poorer performance when participants’ bodies are manipulated to make it harder to ‘reach’ a desired perspective (Cavallo et al., 2017; Deroualle et al., 2015; Kessler & Rutherford, 2010; Kessler & Thomson, 2010; Martin et al., 2020; Surtees et al., 2013; Yu & Zacks, 2017). For example, Deroualle and colleagues found that adults in a Virtual Reality environment took longer to ‘throw’ a ball from one avatar’s location to another’s if the chair they were sitting on was rotated away from the desired throwing location, suggesting that vestibular signals produced by the real movement of the chair interfered with the process of imagining alternative visuospatial relationships in the virtual space. Support also comes from participants’ errors; adults sometimes make manual responses consistent with a perspective they have just imagined, contrary to instructions to respond egocentrically (Samuel, Legg, et al., 2019). In their task, which the experiments in the present study are closely modelled on, participants first needed to disambiguate a target number (e.g. a 6 lying on its side) from a distractor (e.g. a 9 lying on its side) in a 2 × 2 grid by taking the perspective of an avatar located on the left or right of the display. Taking the avatar’s perspective was necessary in such cases because the numbers 6 and 9 are only identifiable when one knows which way up they are seen. After target identification, participants then needed to press one of four buttons also arranged in a grid, mapped to each quadrant of the display such that the top right button corresponded to the top right quadrant and so on, but to always do so from the *egocentric* point of view (see the left panel of Figure 2 for an example from the current studies). For example, when the avatar was on the participant’s right and the target was identified in the top left quadrant from the avatar’s point of view, it was necessary to press the *bottom* left button, as this was where the target was from the participant’s own perspective. Instead, participants sometimes pressed the top left button, a response that is incorrect in the context of the instruction to respond egocentrically, but consistent with the recently imagined perspective. This suggests that taking another’s visual perspective can induce unintentionally altercentric manual actions. Such errors likely arose through the process of embodied perspective taking, whereby participants’ motor representations were briefly integrated with that of the recently taken viewpoint. It follows that, if participants on the perspective taking task by Samuel and colleagues do not always respond egocentrically but are sometimes required to make manual responses based on the *avatar’s* perspective, it could be that performance would be even faster on the latter. This is because it should be easier to make manual responses with perspectives we are already ‘in’ than if we need to ‘disconnect’ first to return to *our* perspective before responding. Relatedly, such practice making manual responses according to other spatial frames of reference may reduce the efficiency with which one responds according to one’s own.

**Figure 2.**
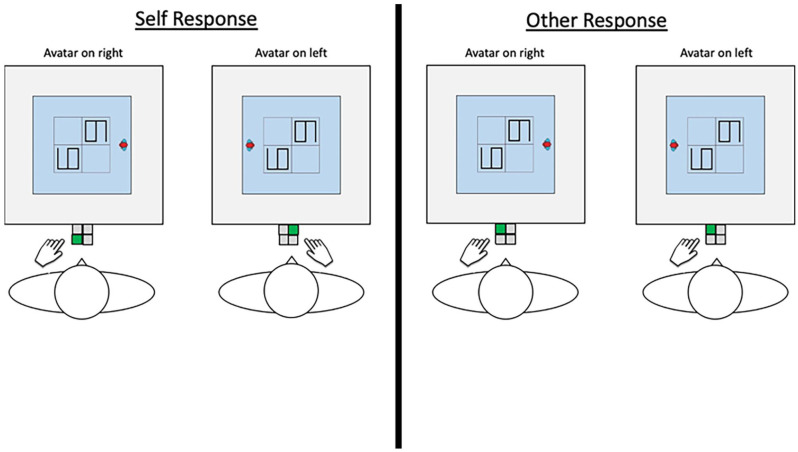
The examples concern trials where the target digit is ‘6’. The correct button is shown in green. Participants respond with the button that corresponds to the target location from their own perspective (self-response trials) or the avatar’s (other-response trials). The left hand was used for the left-sided buttons, the right hand for the right-sided buttons. Note the avatar’s visual perspective is always required to be taken first to locate the correct target.

In sum, previous studies have demonstrated that self-perspective performance declines when other-perspective trials are interleaved, such as in the Dot Perspective task (Samson et al., 2010) and the Director Task (Samuel, Roehr-Brackin et al., 2019). However, the ability to make manual responses consistent with one’s natural spatial frame of reference has not yet been shown to be compromised in this way. We investigated the flexibility of egocentricity in manual action in four experiments. In Experiment 1, we investigated the potential for faster performance on trials from the avatar’s perspective than one’s own. Participants disambiguated a target from a distractor from an avatar’s point of view (e.g. a 6 from a 9), but were then asked to make a manual response mapped to the target’s location from either their own perspective (self-response trials) *or* the avatar’s perspective (other-response trials). Participants performed one block of self-response trials only and one of other-response trials only. Since it was always necessary to consider the avatar’s perspective first to identify the target, all trials are in effect other-*perspective* trials; only the type of response was manipulated (whether participants should press a button consistent with the target location from their own perspective or the avatar’s). We hypothesised that if participants integrate their motor representations with the avatar’s perspective, as embodied perspective taking implies, they should be faster to respond on other-response trials than self-response trials as the latter requires an extra stage of processing to disconnect from the updated reference frame prior to responding. If on the other hand egocentricity in manual action is ‘shielded’ from the effects of taking other perspectives, possibly because of its utility in goal-directed action upon the real world, responses on self-response trials should always be fastest.

## Experiment 1

### Method

#### Participants

Our primary analysis was a comparison between response times (RTs) on self-response trials and other-response trials. A power calculation^1^ using G*Power found that 34 participants were required for an 80% chance to detect a medium effect size (*d* = 0.5). All participants were required to have English as their dominant language (in order to quickly process the auditory cues), be aged 18 to 35 and have normal or corrected-to-normal vision. A minimum 60% accuracy across the task as a whole was required for data inclusion in final analyses (chance accuracy being 25%), as well as 60% accuracy specifically on horizontal-perspective trials, which formed the basis of the primary analysis. This ensured data in the analyses came from participants who understood and followed the instructions. Participants were recruited at the University of Essex and were compensated with course credit. Ethical approval was obtained from the University of Essex Science and Health Ethics Sub-Committee. The final sample included 27 females and 7 males, with a mean age of 19.2 years (range 18–21). The total participation time was approximately 30 min.

#### Materials and procedure

We adopted the task in Samuel, Legg, et al. (2019), as this had already been demonstrated to generate (unintended) manual responses according to alternative perspectives. Participants sat in front of a computer screen laid flat on a table (see Figure 2) situated in the centre of a room large enough for someone to sit at any edge of the screen.^2^ A square white cardboard frame on the screen covered all but a square area of the display large enough to show the grid and avatar (approx. 17 cm square). This was in order to minimise any sense of an intrinsic ‘way up’ for the rectangular screen. Between the screen and the participant was a number pad with the four response keys (5, 6, 8, 9) covered with stickers. Participants were instructed to press the right buttons (6 and 9) and left buttons (5 and 8) with the forefingers of their right and left hands respectively; this manual lateralisation of responses was designed to encourage the embodiment of perspectives (Erle & Topolinski, 2017; Kessler & Thomson, 2010).^3^ The task was designed and presented using EPrime 2.0 software (Psychology Software Tools).

Each trial began with a blank screen and the naming of the target (1,000 ms) via headphones, which was always a pre-recorded ‘six’ or ‘four’ spoken in a female voice (the avatar was described as female). After a further 250 ms of blank screen, an empty 2 × 2 grid appeared for 100 ms followed by the avatar (wearing a red cap, looking towards the grid), the target digit (a 4 or a 6) and the distractor digit, all appearing simultaneously. The two digits were always presented in diagonally opposite squares. The target digit was always upright from the avatar’s perspective. On related condition trials the distractor digit was the target digit but rotated 180 degrees so that it was upside-down. This meant a target 6 was paired with a distractor 9 and a target 4 was paired with an upside-down 4. On unrelated condition trials, the alternative digit was presented instead (a 6 if the target was a 4 and vice-versa), always upright. Related condition trials should be harder because the form of the target and distractor was the same, only the orientation was different. Up to 3,500 ms were allowed for responses, after which a time-out error was recorded. There was then 2,000 ms of blank screen prior to the next trial.

Participants were instructed to locate the target that the avatar named from the avatar’s perspective. How they then responded depended on the block. In the self-response block, the correct response was the target location from their own perspective; in the other-response block, it was the target’s location from the avatar’s perspective. For example, if the avatar was on the left side of the grid and the target was in the top left corner from her perspective, in the self-response block the correct answer would be the top right button (made with the right hand), but in the other-response block it would be the top left (made with the left hand). Crucially, it was always necessary to take the avatar’s visual perspective first to disambiguate the target from the distractor (except perhaps on shared-perspective trials, which in any case were not included in the main analyses – see below).

Before performing the task, participants completed 12 practice trials in which the instruction was simply to press the button that corresponded to the location of a single stimulus (a ‘+’ sign, no distractor) in the grid. The experimenter presided over the practice session and checked that participants used the correct finger, reminding them to do so if they did not. Following practice, participants were given written instructions for the main task, which included the requirement for self- or other-responses. All were required to show the experimenter which button they would press for three example scenes, one where the avatar appeared on the left, one on the right and one where the avatar was at the bottom, sharing the participant’s point of view. If the participant did not respond correctly, further examples and clarification were provided until the participant did. The same examples were again provided before the second block, and the participant was required to demonstrate that they now responded according to the new response instruction. Half of the participants performed the self-response block first and the other half the other-response block first, and the instructions at the start matched the required response system for each block.

Each block contained 64 randomly presented trials, equally divided into left-perspective, right-perspective, shared-perspective and opposite-perspective trials. These latter two perspectives were included to ensure that what appeared to be the target from the participant’s own point of view could sometimes be correct (shared-perspective) or misleading (opposite-perspective), making attending to the avatar’s perspective crucial. Data from these perspectives were not however included in our main analysis because on opposite-perspective trials a correct response from the avatar’s perspective is confounded with a distractor error from the self-perspective, and on shared-perspective trials it is not possible to know whether the participants respond according to their own perspective or the avatar’s. The crucial test of our hypothesis thus concerned the horizontal (left and right) perspectives alone, and it was the data from these perspectives that were used in the main analysis. The 16 trials from all four perspectives were equally divided between related and unrelated distractor trials, trials with ‘four’ as a target and trials with ‘six’ as a target and target and competitor location (across all four grid squares).

#### Transparency and openness

We pre-registered the hypothesis, methods and analyses for this study, the details of which can be found here: https://osf.io/v7582. The data can be found here: https://osf.io/8ujce/files/osfstorage.

### Results

#### Confirmatory analyses

##### Accuracy

A related-samples Wilcoxon Signed-Rank test found no statistically significant difference between accuracy on the self-response block (Mdn = 94%) and accuracy on the other-response block (Mdn = 94%), *W*(34) = 209, *p* = .86. Median accuracy was in fact identical across the two perspectives.

##### Response times

There was no statistically significant difference between mean RTs on self-response trials (M = 1,599 ms) and other-response trials (M = 1,596 ms), *M*_Diff_ = 3 ms (95% CI [−5, 7]), *t*(33) = 0.155, *p* = .88, *d* = 0.058. A Bayesian paired-sample *t*-test^4^ found that the data were five times more likely under the null than both (a) an alternative hypothesis positing faster RTs for other-response trials (*BF*_10_ = 0.190) and (b) an alternative hypothesis simply that the two means differed (*BF*_10_ = 0.184). In sum, contrary to our hypothesis participants were not faster on other-response trials than self-response trials. However, they were also not faster on self-response trials than other-response trials.

#### Exploratory analyses

**Figure 3.**
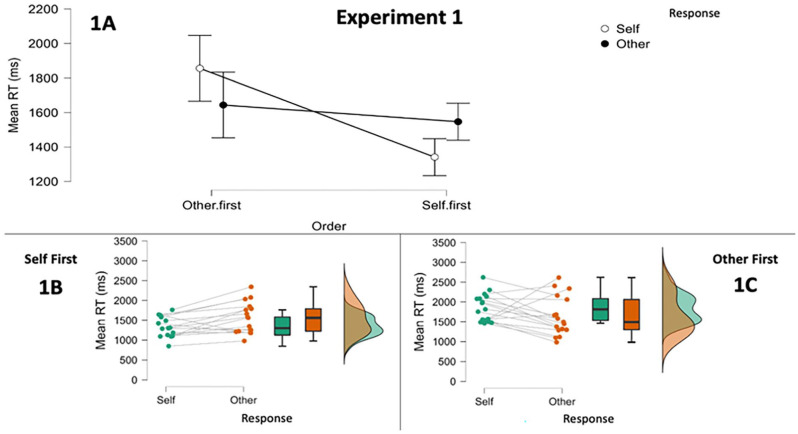
Results of Experiment 1. Panel 1A displays the condition means and 95% confidence intervals for the analysis including block order. Panels 1B and 1C show the mean RTs for each participant and for each response instruction as a function of block order. *Note*. RT = response time.

Our main analysis had been collapsed over the order in which participants performed the task (self- or other-response block first). Reasoning that this might mask important differences, we conducted a 2: Order (Self First vs. Other First) × 2: Response (Self RT vs. Other RT) mixed-design ANOVA. As before, the analysis included only trials where the avatar was on the left or right of the display. Results are displayed in Figure 3. The analysis revealed a main effect of Order, *F*(1, 32) = 7.938, *p* = .008, $\eta_{p}^{2}$ = .199, with the group that performed other-perspective trials first 306 ms slower overall (95% CI [85, 527]). There was no main effect of Response, *F*(1, 32) = 0.002, *p* = .97, $\eta_{p}^{2}$ = 0. There was however a significant interaction which appeared to show that the egocentric advantage when self-response trials were performed first disappeared when these were performed second, *F*(1, 32) = 8.232, *p* = .007, $\eta_{p}^{2}$ = .205.

This was confirmed by follow-up post-hoc analyses. The group who performed self-response trials first were faster on these than on other-response trials, *M*_Diff_ = 206 ms, 98.75% CI [3, 409], *t*(16) = 2.871, *p* = .011 (corrected alpha = .0125), with a medium-to-large effect size *d* = 0.696. In contrast, those who performed the other-response block first were not reliably faster on either block, *M*_Diff_ = 212 ms, 98.75% CI [−147, 572], *t*(16) = 1.673, *p* = .11 (corrected alpha = .0125), *d* = 0.406.^5^ Between-group comparisons found that RTs on other-response trials did not vary according to order, *M*_Diff_ = 97 ms, 98.75% CI [−310, 503], *t*(32) = 0.634, *p* = .53 (corrected alpha = .0125), *d* = 0.22, but RTs on self-response trials were faster in the group who performed these trials first, *M*_Diff_ = 515 ms, 98.75% CI [237, 794], *t*(32) = 4.924, *p* < .001 (corrected alpha = .0125) and with a large effect size *d* = 1.69.

In accuracy, those who performed self-response trials first performed similarly on both self-response (Mdn = 97%) and other-response trials (Mdn = 94%), *W*(17) = 80.5, *p* = .08 (adjusted alpha = .025). For those who performed other-response trials, first accuracy was also similar on both self-response (Mdn = 88%) and other-response trials (Mdn = 94%), *W*(17) = 34, *p* = .15 (adjusted alpha = .025).

### Discussion

We gave participants a perspective taking task in which a target digit needed to be located from an avatar’s perspective and then a manual response made that was consistent with either the participant’s self-perspective or the recently imagined, other-perspective. We hypothesised that since participants should integrate their motor representations with the avatar’s perspective first in order to identify the target, they would be faster to make a manual response consistent with that perspective than their own, which would require the extra step of ‘returning’ to one’s own manual frame of reference. Results did not support this hypothesis. However, they did show that egocentric bias was eliminated. This is because regardless of the order in which participants performed the task, RTs were by the end equivalent across both self- and other-response trials. Additionally, exploratory tests found that the egocentric speed advantage for self-response trials was present when self-response trials were performed first but *disappeared* when other-response trials were performed first. RTs on other-response trials were broadly consistent regardless of when they were performed. Such an effect is consistent with previous research in VPT showing the elimination of an egocentric speed advantage following an other-perspective trial (Samuel, Roehr-Brackin, et al., 2019). These results extend the elimination of egocentricity to manual action, as participants were no faster on self-response trials than other-response trials when response buttons were spatially mapped to their own or the avatar’s perspectives. They also extend the duration of the effect beyond a single trial to a 64-trial block approximately 8 to 10 min long. However, since the results of Experiment 1 came primarily from exploratory analyses we conducted a second experiment in an attempt to replicate and extend this finding.

## Experiment 2

### Method

Following from the outcome of Experiment 1, in Experiment 2, we hypothesised that participants who received the self-response block first would be faster on self-response trials than other-response trials, but that participants who performed other-response trials first would not show this egocentric advantage. In line with our original hypothesis, we also allowed that any egocentric speed advantage in the former group could yet *reverse* for the latter participants. This is because the majority of participants in this group in Experiment 1 patterned in this direction, with only a minority of participants patterning more strongly in the opposite direction (see Figure 3, panel 1C). We hypothesised that this pattern should also be evidenced in an identical analysis of a subset of data made up from related condition trials only, as in these trials alternatives to perspective taking such as form-based strategies for locating targets are not available. Except where noted, no changes were made from Experiment 1.

#### Participants

A new sample was recruited at the University of Plymouth and was compensated with course credit. Ethical approval was obtained from the University of Plymouth Faculty of Health Research Ethics and Integrity Committee. Although we pre-registered an *N* of 34, we obtained data from 36 participants after advertising for four replacement participants for failing to meet the minimum accuracy criteria of 60% led to an accidental over recruitment. We retained the full sample of 36 for the increase in statistical power. The final sample included 31 women and 5 men, with a mean age of 19.6 years (range 18–24). The total participation time was approximately 30 min.

#### Materials and procedure

The experiment was now run using Open Sesame version 3.3.9, and the 12 practice trials were now as experimental trials but with feedback (green screen for a correct response, red screen for an incorrect response). Practice trials were only presented once, at the beginning of the first block. For the second block and instructions switch, participants were shown example grids on paper and asked to show which button they would press given the new instructions, as before. Once the experimenter was satisfied that the new response method was understood the second block began.

#### Transparency and openness

As before, we pre-registered the hypothesis, methods and analyses for this study, the details of which can be found here: https://osf.io/n8gpt. The data can be found here: https://osf.io/8ujce/files/osfstorage.

### Results

#### Confirmatory analyses

##### Main analyses

We predicted that analyses would show an interaction suggesting that self-response trials would be performed more quickly than other-response trials when the self-response block came first, while performance on other-response trials would either remain equivalent across the order factor or even reverse, meaning faster performance on other-response trials than self-response trials when the other-response block was performed first. This should be borne out by a combination of between- and within-subjects planned follow-up analyses.

**Figure 4.**
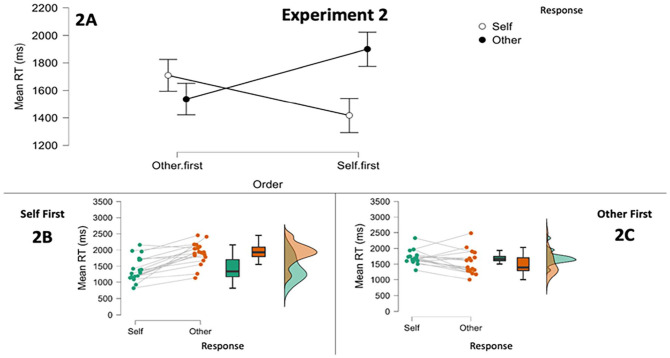
Results of Experiment 2. Panel 2A displays the condition means and 95% confidence intervals for the analysis including block order. Panels 2B and 2C show the mean RTs for each participant and for each response instruction as a function of block order. *Note*. RT = response time.

Initial analyses found the mean RT data deviated from normality in one cell. However, a log transformation led to deviation in a different cell. We, therefore, conducted a 2: Order (Self First vs. Other First) × 2: Response (Self RT vs. Other RT) mixed-design ANOVA on untransformed RTs. As before, the analysis only included trials where the avatar was on the left or right of the display. Results are displayed in Figure 4. The analysis revealed a main effect of Perspective, *F*(1, 34) = 7.380, *p* = .01, $\eta_{p}^{2}$ = .178, with other-response trials 154 ms slower overall (95% CI [39, 270]). There was no main effect of Order, *F*(1, 34) = 0.137, *p* = .71, $\eta_{p}^{2}$ = 0. However, and crucial to our hypothesis, there was a significant interaction, *F*(1, 34) = 33.097, *p* < .001, $\eta_{p}^{2}$ = .493. The interaction appeared to signal that the egocentric advantage in RTs not only disappeared but also reversed if self-response trials were performed second.

Planned independent *t*-tests (Bonferroni-corrected) revealed that RTs on self-response trials were faster when they were performed first than second, *M*_Diff_ = 292 ms, 98.75% CI [22, 563], *t*(34) = 2.870, *p* = .007 (corrected alpha = .0125), with a large effect size *d* = 0.957.^6^ Conversely, RTs on other-response trials were faster when these were first, *M*_Diff_ = 362 ms, 98.8% CI [49, 675], *t*(34) = 3.073, *p* = .004 (corrected alpha = .0125), again with a large effect size *d* = 1.02. In sum, self-response trials were performed faster when the self-response block came first, and other-response trials were performed faster when the other-response block came first. Planned analyses at the within-group level found that the group who performed self-response trials first were faster on that block, *M*_Diff_ = 482 ms, 98.75% CI [247, 717], *t*(17) = 5.764, *p* < .001 (corrected alpha = .0125), *d* = 1.358. This confirmed the egocentric advantage in those who performed self-response trials first, with a large effect size. Those who performed the other-response block first were just as quick on these as self-response trials, *M_Diff_* = 173 ms, 98.75% CI [−44, 390], *t*(17) = 2.238, *p* = .039 (corrected alpha = .0125), *d* = 0.528. This again confirmed the elimination of egocentricity (with a medium effect size).

Accuracy was high, with percentage correct scores exceeding 85% in all conditions. The pattern of results in RTs was reflected in accuracy analyses. Accuracy was higher on the self-response block when it was performed first (Mdn = 97%) than second (Mdn = 91%), *W*(36) = 78, *p* = .007, and higher on the other-response block when that was performed first (Mdn = 99%) than second (Mdn = 94%), *W*(36) = 229, *p* = .031. In those who performed the self-response block first, accuracy was higher on self-response trials (Mdn = 97%) than other-response trials (Mdn = 91%), *W*(18) = 102.5, *p* = .017. In those who performed the other-response block first, accuracy was higher on other-response trials (Mdn = 99%) than self-response trials (Mdn = 91%), *W*(18) = 20, *p* = .044.

##### Analyses of a subset (related condition trials)

As pre-registered, we also ran the same analyses using a subset formed of data from related condition trials only, where the distractor was identical to the target but inverted. A 2: Order (Self First vs. Other First) × 2: Response (Self RT vs. Other RT) revealed no main effect of Perspective, *F*(1, 34) = 1.563, *p* = .22, $\eta_{p}^{2}$ = .044, or Order, *F*(1, 34) = 0.042, *p* = .84, $\eta_{p}^{2}$ = 0, but the crucial interaction was replicated, *F*(1, 34) = 28.070, *p* < .001, $\eta_{p}^{2}$ = .452. This again signalled that the egocentric speed advantage when self-response trials were performed first reversed if self-response trials were performed second. The results of planned independent *t*-tests (Bonferroni-corrected) were largely consistent with the main analyses, revealing that RTs on self-response trials were marginally faster when they were performed first than second, *M*_Diff_ = 310 ms, 98.75% CI [−3, 624], *t*(34) = 2.629, *p* = .013 (corrected alpha = .0125), with a large effect size *d* = 0.876.^7^ RTs on other-response trials were faster when these were first, *M*_Diff_ = 352 ms, 98.8% CI [34, 669], *t*(34) = 2.942, *p* = .006 (corrected alpha = .0125), again with a large effect size *d* = 0.981. Planned analyses at the within-group level found that the group who performed self-response trials first were faster on that block, *M*_Diff_ = 409 ms, 98.75% CI [119, 700], *t*(17) = 3.962, *p* = .001 (corrected alpha = .0125), with a large effect size *d* = 0.934. Those who performed the other-response block first were faster on these instead, *M*_Diff_ = 253 ms, 98.75% CI [55, 451], *t*(17) = 3.595, *p* = .002 (corrected alpha = .0125), with a large effect size *d* = 0.847. This result represented the first statistically significant demonstration of faster performance on other-response trials, that is, a reversal of egocentricity. Accuracy was high, with percentage correct scores exceeding 84% in all conditions. The pattern of results in RTs was reflected in accuracy analyses. Accuracy was higher on the self-response block when it was performed first (Mdn = 100%) than second (Mdn = 94%), *W*(36) = 101, *p* = .044, and higher on the other-response block when that was performed first (Mdn = 100%) than second (Mdn = 91%), *W*(36) = 244.5, *p* = .006. In those who performed the self-response block first, accuracy was higher on self-response trials (Mdn = 100%) than other-response trials (Mdn = 91%), *W*(18) = 87, *p* = .031. In those who performed the other-response block first, accuracy did not differ between other-response trials (Mdn = 100%) than self-response trials (Mdn = 94%), *W*(18) = 15.5, *p* = .13.

### Discussion

Across the experiment as a whole, participants were faster on self-response trials than other-response trials, supporting the egocentric advantage and contrary to the possibility that it could be eliminated. However, the results of Experiment 2 not only confirm the elimination of egocentricity through practice responding according to alternative perspectives but also support the reversal of egocentricity on related condition trials. Specifically, while RTs were faster and accuracy higher on self-response trials than other-response trials whenever self-response trials came first, speed on other-response trials did not differ from speed on self-response speed, and was sometimes faster on *other*-response trials, whenever other-response trials came first. The elimination of egocentricity in RTs became a reversal when the requirement to disambiguate stimuli was at its highest (on related condition trials). In sum, adults can respond equally well and even better from alternative perspectives than their own after only a few minutes of practice responding according to the other point view.

## Experiment 3

In Experiment 3, we investigated performance in a version of the task in which both self- and other-response trials were performed in a mixed rather than blocked design. Now participants had to respond either according to their own or to the avatar’s perspective as cued on a *trial-by-trial basis*. We did this to test whether the overall elimination of egocentricity across the task as a whole, which was found in Experiment 1 but not in Experiment 2, would occur when the number of self- and other-response trials were approximately the same at all points in the task. We hypothesised that mixing perspectives in this way would weaken the egocentric default sufficiently to again eliminate the egocentric advantage in manual responding at the level of the Experiment as a whole (i.e. performance on self-response trials should not be faster or more accurate than performance on other-response trials). Support for this hypothesis would indicate that a block of exclusively other-response trials is not the only means of eliminating egocentricity, but also a running 50/50 split of self- and other-response trials. In line with some of the results from Experiment 2, we also tentatively hypothesised that the egocentric advantage might not only be eliminated but also reverse, that is, other-response trials could be faster and/or more accurate than self-response trials. As in the previous two experiments, the participant always needed to disambiguate the target from the distractor from the avatar’s perspective *first*; the terms ‘self’ and ‘other’ perspective refer to the type of *response* required. Each response type was still required for half of the total number of trials in the task. Experiment 3 also gave us the opportunity to investigate performance at the trial level, such as the impact of switching response types compared to repeating them. Previous research has shown that VPT performance is usually diminished on trials which require a perspective switch relative to a perspective repeat (Chiu et al., 2019; Ferguson et al., 2017; Samuel, Roehr-Brackin, et al., 2019). Finally, results from Experiments 1 and 2 suggested an overall trend towards slower performance on the second block, irrespective of which perspective came second. Although a chronological order effect cannot explain the crucial interaction found in each of those experiments, the mixed design in Experiment 3 allowed us to test whether the elimination of egocentricity would occur when block was removed entirely. Experiment 3 was considered exploratory and was not pre-registered.

### Method

#### Participants

In order to increase the power of the study, we collected data from twice the number of participants as we did for Experiments 1 and 2. We recruited 116 participants in total, 6 of whom were later excluded for technical failures and a further 43 for failing to achieve a minimum accuracy rate (see *analyses* below). Participants were paid rather than given course credit. The final sample of 67 participants included 39 females, 26 males and 2 non-binary individuals, with a mean age of 23 years (range 18–35). The total participation time was approximately 15 min. All were recruited from the University of Essex. Ethical approval was obtained from the University of Essex Science and Health Ethics Sub-Committee.

#### Materials and procedure

The procedure was identical to Experiment 1 with the following exceptions. Firstly, participants were always tasked with finding the target ‘6’. The distractor was always a ‘9’ (there were no ‘4’s or upside-down 4s). This allowed for a new audio cue which informed the participant which perspective to respond from on a trial-by-trial basis (‘you’ or ‘her’).

Secondly, participants performed a single block of 84 trials. The first four of these were excluded. The 80 remaining trials were equally divided into self- and other-response trials, avatar location (left and right) and repeat trials (response from the same perspective consecutively) and switch trials (response from the perspective that was not used in the previous trial). The trial sequence was pseudo-randomly fixed to create equal numbers of self-SELF, other-OTHER, self-OTHER and other-SELF trials, where the word in capitals describes the target trial and the word in lower case letters describes the preceding (*n−*1) trial. There were thus 20 analysable trials of each of these four sequences.

Thirdly, shared- and opposite-perspective trials were removed from the task, leaving only left- and right-sided perspectives of the grid. These were the only trial types analysed in the previous two Experiments, and removing them helped to keep the task at approximately the same duration.

Fourthly, participants now performed the task sat at a table with an upright computer screen rather than one laid flat. This is because the results of another study using this task found that the ability to take other perspectives around the grid was not impacted by the accessibility or otherwise of the space around it (Samuel et al., 2023). Since shared and opposite-perspective trials were removed in Experiment 3, participants performed the task looking at a screen set up in the usual ‘landscape’ orientation as neither perspective was privileged by doing so.

Finally, the inter-trial interval was reduced in length from 2s to 1s. The added difficulty in the speed of the task and the requirement to switch perspectives on a trial-by-trial basis was designed to be partially offset by the use of only a single target throughout (6) and the absence of shared- and opposite-perspective trials, which removes not only the hardest perspective type from the task but also means digits never appeared in their most distracting, canonical form. However, the high number of participants whose data were excluded for failing to achieve the minimum accuracy thresholds suggests this was a particularly difficult version of the task.

Prior to the experimental block of trials, participants performed 16 practice trials, all of which were randomly presented. Half were self-response and half were other-response. Feedback on each response was provided immediately and by the program itself (the screen turned green for a correct response, or red for an incorrect response or a timeout).

#### Analyses

Given the introduction of inter-trial perspective switches and a shorter time window for responses, we expected slightly lower accuracy in Experiment 3 than Experiment 2. We set 50% accuracy across the task (and at least 40% for each of the left and right perspective trials) as a minimum for inclusion in the final analyses. Given that chance accuracy is 25%, scores lower than these rates would suggest that the participant failed to follow the instructions or simply found the task too difficult conceptually or performatively.^8^ The test of our hypothesis was as follows; if the egocentric bias in manual responding is eliminated with practice responding according to imagined perspectives, performance should be no better on self-response trials than other-response trials. This would be measured by comparing self-response and other-response trials in both accuracy and speed, with speed considered the more informative measure owing to its sensitivity. Additionally, the absence of a difference between self- and other-response performance should be at least three times more likely under this null hypothesis than the alternative hypothesis, measured by Bayesian analyses (Dienes, 2014). Finally, further exploratory analyses would be conducted to look for any interesting patterns in trial-by-trial performance.

#### Transparency and openness

Experiment 3 was not pre-registered. The data can be found here: https://osf.io/8ujce/files/osfstorage.

### Results

#### Accuracy

**Figure 5.**
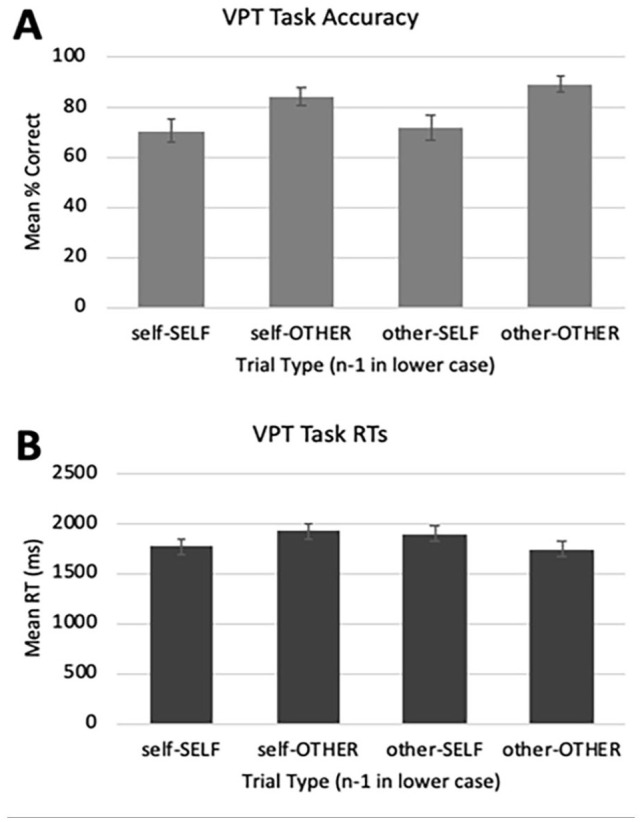
Results of Experiment 3. Panel A displays mean accuracy (%), Panel B mean response times (ms). Error bars represent 95% confidence intervals.

Figure 5A displays mean accuracy by trial type. Overall accuracy in the group that were retained following data cleaning was high but not at ceiling (*M* = 78.8%, range = 53.8%–100%, *SD* = 13.4%). Shapiro–Wilks tests found the distribution of accuracy scores deviated from normality, and we thus proceeded with non-parametric testing. A Wilcoxon Signed-Rank test found that participants were in fact 18% more accurate on *other*-response trials (Mdn = 90%, 95% CI [84%, 89%]) than self-response trials (Mdn = 72%, 95% CI [66%, 76%]), *W*(67) = 1,565, *Z* = 5.495, *p* < .001, *r* = .475. Egocentricity in accuracy was thus not only eliminated but reversed.

#### Response times

Figure 5B displays the mean RTs by trial type. Shapiro–Wilks tests found no evidence that the distribution of RT data deviated from normality. Consistent with our hypothesis that egocentricity would be eliminated, a paired-sample *t*-test found no evidence that mean RTs on self-response trials (M = 1,836 ms, 95% CI [1,759, 1,913]) differed from mean RTs on other-response trials (M = 1,828 ms, 95% CI [1,752, 1,903]), *t*(66) = 0.220, *p* = .83, *d* = .027. A post-hoc power analysis conducted using G*Power (3.1) found a 58% chance to detect an effect of this size with 67 participants. A Bayesian paired-sample *t*-test supported the null, finding these data were approximately seven times more likely under the null hypothesis that there is no difference between perspectives (*BF*_10_ = 0.137).

#### Exploratory analyses

Next, we conducted an exploratory test of the effect of switching between perspectives on accuracy and RTs. A non-parametric Friedman’s related-samples two-way ANOVA by ranks found that accuracy across the four sequences differed, with a chi-square value of 59.082, *p* < .001. Pairwise contrasts using the Bonferroni correction found participants were more accurate on other-OTHER trials than every other trial type (self-OTHER: *W*(67) = 0.597, *Z* = 2.677, adj. *p* = .007, *r* = .231; other-SELF: *W*(67) = 1.313, *Z* = 5.889, adj. *p* < .001, *r* = .509; self-SELF: *W*(67) = 1.403, *Z* = 6.290, adj. *p* < .001, *r* = .543). They were also more accurate on self-OTHER trials than both self-SELF, *W*(67) = 0.806, *Z* = 3.613, adj. *p* < .001, *r* = .312 and other-SELF, *W*(67) = 0.716, *Z* = 3.212, adj. *p* = .001, *r* = .277. No difference was found between other-SELF and self-SELF trials, *W*(67) = 0.090, *Z* = 0.401, adj. *p* = 1, *r* = .035. Overall, accuracy was always higher on other-perspective trials than self-perspective trials, again supporting a reversal in egocentricity.

We ran a similar analysis for RTs. A 2: Response (Self vs. Other) × 2: Switch Type (Non-Switch vs. Switch) fully within-subjects ANOVA found a significant main effect of Switch Type, *F*(1,66) = 74.149, MSE = 21,269, *p* < .001, $\eta_{p}^{2}$ = .529. As expected, participants were 153 ms slower on Switch trials (M = 1,911 ms, 95% CI [1,841, 1,981] than on Non-Switch trials (M = 1,757 ms, 95% CI [1,689, 1,826]. There was no main effect of Response, *F*(1,66) = 2.085, MSE = 19,293, *p* = .15, $\eta_{p}^{2}$ = .031; participants performed at similar speeds across self-response trials (M = 1,836 ms, 95% CI [1,759, 1,913]) and other-response trials (M = 1,828 ms, 95% CI [1,752, 1,903]. There was no significant interaction, *F*(1,66) = 0.003, MSE = 88,441, *p* = .96, $\eta_{p}^{2}$ = .003, suggesting switching was equally difficult whether it was to an other-response trial or to a self-response trial.

### Discussion

The results of Experiment 3 demonstrate again that egocentricity can be eliminated. Whereas in Experiments 1 and 2, this was found after a block of other-response trials, here it was found when response types were mixed and no one response type was at any given point significantly more practised than the other. Participants were also more accurate on *other*-response trials than self-response trials. This suggests that at the level of accuracy, at least, egocentricity might not merely be eliminated but can reverse. However, there was no evidence pointing to a specific effect on egocentricity when it came to trial-by-trial switching; participants were slower on switch trials than repeat trials but this slowdown did not vary significantly by the direction of the switch (self to other or other to self). Intriguingly, this suggests that when looking at trial-by-trial performance in a mixed-block design, egocentricity is no less malleable than its altercentric counterpart.

Overall, Experiments 1 to 3 had so far shown that egocentricity is eliminated after a block of other-response trials and when response types are mixed within a single experiment. There was some evidence of the reversal of egocentricity, but in each instance where this occurred it was within a specific subset of data (related condition trials in Experiment 2, in accuracy alone in Experiment 3), suggesting reversal is limited. In Experiment 4, we sought to better understand what makes egocentricity deteriorate.

## Experiment 4

An important question that follows from these findings concerns whether this modulation of egocentricity occurs through the practice making manual responses consistent with other perspectives even in the absence of any requirement to understand how things appear visually from different perspectives. This could occur through practice with non-egocentric S-R mappings. For example, in the (visual) Simon task (Hommel, 2011), participants make a spatial response to the colour of a stimulus, such as a left key for a red square and right key for a blue square, whilst trying to ignore the irrelevant spatial dimension of the stimulus (appearing on either the left or right side of the screen). The spatial incongruence of the stimulus location and the required response leads to a delay relative to congruent trials, known as the Simon Effect. The Simon Effect or its equivalent on similar tasks can be reduced, eliminated and even reversed if participants first spend time making incompatible responses, such as a left button for a right stimulus and a right button for a left stimulus (D’Ascenzo et al., 2021; Proctor & Lu, 1999; Tagliabue et al., 2000; Xu et al., 2022). Thus one possibility is that the elimination of egocentricity in the experiments here has nothing to do with the visual component of perspective taking but is instead the result of practice with alternative S-R mappings. We examined this question with a fourth experiment in which participants were shown not 6s and 4s but a square and a cross. Since these shapes are visually identical from all four angles, participants no longer need to represent the avatar’s perspective to disambiguate the target from the distractor; they only need to map the target location within the desired perspective in order to press the correct button on other-perspective trials. As such, the manual response requirements for other-perspective trials in Experiment 4 were identical to those of all previous experiments, but now there was no need to infer *visual* information (what the target appeared to *be* from another perspective), only *spatial* information (*where* the target was in the grid from another perspective). If practice with non-egocentric stimulus-response mappings is enough to eliminate egocentricity, then the egocentric advantage should also disappear after a block of other-response trials even with these simpler stimuli, just as it had in the earlier blocked versions of the task (Experiments 1 and 2). If on the other hand, the egocentric advantage persists even after a block of other-response trials, then the requirement to disambiguate the target from alternative viewpoints (i.e. VPT) is a better explanation for the elimination of egocentricity found in the previous experiments.

### Method

In Experiment 4, we returned to the blocked format of Experiments 1 and 2 and tested whether simply making manual responses that were incongruent with the egocentric spatial frame of reference would be enough to produce the elimination and/or reversal the egocentric advantage in RTs found in the previous experiments. To this end, participants no longer needed to use visual perspective information to disambiguate targets, as they were symmetrical shapes (squares and crosses – see Figure 5, panel 4A). Participants located the target and made a manual response consistent with its location from either the self- or other-perspective, as before. The previous audio cues for the digit stimuli were replaced with ‘square’ and ‘cross’, as before in a female voice. Given the new stimuli, there was no longer a related/unrelated stimulus distinction. No other changes were made from Experiment 2.

#### Participants

A new sample was recruited at City St. George’s, University of London. All were compensated with course credit. Ethical approval was obtained from the City St. George’s Research Ethics Committee. Two original participants failed to meet the minimum accuracy criteria of 60% on horizontal perspective trials and these were replaced. The final sample included 32 women and 2 men, with a mean age of 19.3 years (range 18–30). Total participation time was approximately 30 min.

#### Transparency and openness

We pre-registered the hypothesis, methods and analyses, the details of which can be found here: https://osf.io/ghbtc. The data can be found here: https://osf.io/8ujce/files/osfstorage.

### Results and discussion

As we were mainly interested in RTs, no confirmatory analyses of accuracy were pre-registered. For descriptive purposes, mean accuracy was high on self-response trials (self-response trials first *M* = 99.9%, other-response trials first *M* = 97.9%) and on other-response trials (other-response trials first *M* = 86%, self-response trials first *M* = 90.4%).

#### Confirmatory analyses

##### Main analyses

**Figure 6.**
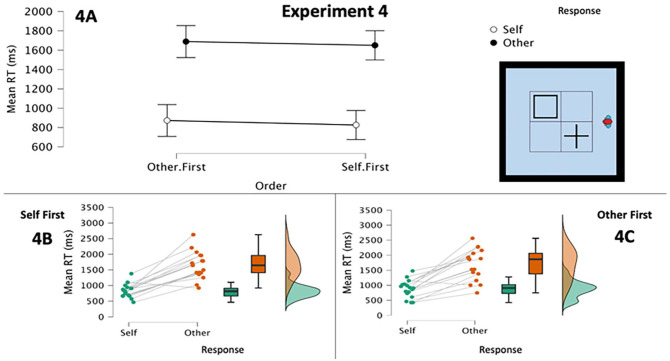
Results of Experiment 4. Panel 4A displays an example grid on the right and on the left the graph shows the condition means and 95% confidence intervals for the analysis including block order. Panels 4B and 4C show the mean RTs for each participant and for each response instruction as a function of block order. *Note*. RT = response time.

As the data in each cell were normally distributed we proceeded with a 2: Order (Self First vs. Other First) × 2: Response (Self RT vs. Other RT) mixed-design ANOVA on mean RTs. As before, the analysis included only trials where the avatar was on the left or right of the display. Results are displayed in Figure 6. The analysis revealed a main effect of Response, *F*(1, 32) = 120.772, *p* < .001, $\eta_{p}^{2}$ = .791, with other-response trials 820 ms slower overall (95% CI [670, 969]). There was no main effect of Order, *F*(1, 32) = 0.155, *p* = .70, $\eta_{p}^{2}$ = 005. Crucially, there was also no interaction, *F*(1, 32) = 0.003, *p* = .96, $\eta_{p}^{2}$ = 0. In sum, participants were slower on other-response trials than self-response trials and performing the other-response trials first did not speed up (or slow down) responses. Contrasted with the results from Experiments 1 to 3, this suggests that the locus of the elimination and reversal of egocentricity in those experiments is the disambiguation of stimuli from other perspectives and not practice responding according to different reference frames.

##### Post-hoc analyses

We conducted a post-hoc Bayesian ANOVA to look more closely at this null interaction and found that the data were eight times more likely under the null hypothesis that there is no interaction (*BF*_10_ = 0.125).

## General discussion

Previous research has shown that taking other visual perspectives can reduce the salience of one’s own, such that the usual speed advantage for self-perspective trials is eliminated if it comes immediately after an other-perspective trial (Samuel, Roehr-Brackin, et al., 2019). In Experiment 1 of the present study, we hypothesised that the requirement to take another agent’s visual perspective first (to disambiguate a target from a distractor) would privilege subsequent manual actions consistent with that perspective such that manual responses made from a first-person perspective would be slower overall than other-response trials. That is, egocentric bias should reverse. This could occur because participants integrate their perspective with the other-perspective (i.e. embodiment), making responding according to the other-perspective easier than responding according to the self-perspective. We found no evidence to support a reversal but did find that self-response trials were not faster than other-response trials, contrary to typical performance on perspective taking tasks. Exploratory tests also revealed an order effect; participants’ speed on self-response trials varied according to whether they began with self-response trials (faster) or other-response trials (slower). We formed a new hypothesis based on this result, namely that the self-response speed advantage would disappear and perhaps reverse in participants who performed other-response trials before self-response trials. That is, practice responding according to the avatar’s perspective should eliminate the egocentric advantage, though not necessarily reverse it. Experiment 2 confirmed this hypothesis; participants were faster on self-response trials when a block of these trials came first but performed equivalently on both self- and other-response trials when the latter came first. In the case of related stimuli, where the distractor had the same form as the target, participants who performed other-response trials first were in fact slowest on self-perspective trials, demonstrating a reversal of egocentricity in this subset of data.

Experiment 3 again confirmed the elimination of egocentricity, this time over the course of a mixed-block design. Accuracy results from Experiment 3 also suggested that it was easier to respond according to the avatar’s perspective than one’s own under these mixed conditions. However, it was not yet clear whether the elimination of egocentricity occurred because it was necessary to take another visual perspective or because of practice making responses according to that perspective. In Experiment 4, we used symmetrical stimuli to remove the requirement to disambiguate stimuli visually. Now, performance was entirely agnostic to whether self- or other-response trials came first. Taken together, these experiments suggest that the elimination and occasional reversal of egocentricity arises specifically through the requirement to *disambiguate* a target from a distractor from an alternative perspective. These results build upon previous findings of flexibility in the egocentric default in perspective taking, extending the duration of the effect to multiple trials (and minutes) after the last-performed other-response trial and provide the first evidence that one’s own, first-person perspective of the world can be so compromised as to cause egocentric bias to reverse. Given that egocentricity in manual action is usually advantageous for successful, goal-directed action in the real world, this is particularly striking.

The results of Experiment 4, differing as they do from Experiments 1 to 3, imply a qualitative change in how participants performed that version of the task. We think that the most plausible explanation for this difference is the recruitment or not of embodiment perspective taking. In Experiments 1 to 3, participants should have performed a mental transformation of their frame of reference, integrating their motor representations with the target perspective. Embodied perspective taking is commonly thought to arise through what is often termed Level 2 perspective taking (Flavell et al., 1981; Kessler & Thomson, 2010; Masangkay et al., 1974), namely those problems concerning the relative appearance or spatial location of an object. This is because this type of task is less amenable to ‘simpler’ solutions such as line-of-sight reasoning (Michelon & Zacks, 2006; for a review see Samuel, Cole, et al., 2022). Both of these problems were found simultaneously in Experiments 1 to 3; the target digit had to be disambiguated through a judgement about relative appearance and on other-response trials the target’s relative spatial location had to be used to make a correct response. Embodiment is also what the types of errors committed on a previous version of this task has strongly implied, as outlined in the Introduction (Samuel, Legg, et al., 2019). If correct, this repeated practice of embodied perspective taking over multiple trials could have led to a ‘blending’ of first-person and second-person representations, eroding the egocentric default. Alternatively, the repeated other-response trials could have engaged inhibitory processes upon first-person representations. This inhibition would need to remain in place long enough for the impact of the first block of trials to persist for much of the second. Either way, the duration of this effect, lasting as it did even several minutes after the last self-perspective trial, represents a significant leap from the single-trial effect previously reported (Samuel, Roehr-Brackin, et al., 2019).

In contrast, in Experiment 4, relative appearance was not a factor given that the stimuli were identical from all four perspectives in the task. However, extracting the relative spatial location code of the target *was* still necessary on other-perspective trials, and thus embodiment and subsequent elimination of egocentricity might still have been expected to arise, as it would be useful to integrate what is on the avatar’s left/right upper/lower segments in order to make these responses. Instead, it is likely that in Experiment 4, participants did not engage embodied processes. They may have developed a simple heuristic whereby the target was first identified egocentrically (a trivially easy task given the stimuli) and then triangulated with the avatar’s location. A spatial code could therefore be generated for a successful response, but importantly this would be achieved via geometric reasoning rather than an inference of the *content* of a perspective. Crucially, in Experiments 1 to 3, any such heuristic would have been more difficult to develop owing to the nature of the stimuli. On unrelated trials, where 6 and 4 were the contrasting stimuli, it would have been very difficult (though not impossible) to identify the target through any form-based strategies alone, making the consideration of perspective very likely. On related trials, where the identity of the digits (6 and 9) pivoted entirely on the perspective from which they were viewed, such heuristics would have been useless. Indeed, it is on related trials alone, in Experiment 2, that egocentric bias clearly reversed. This appears to strengthen the possibility that it is the disambiguation of stimuli that elicits embodied transformations.

An alternative source of the elimination of egocentricity, though one which we feel is less likely, could be found in the generation of a mental image corresponding to the target perspective. That is, if participants represented the visual experience of the alternative perspective in the form of a mental image and integrated this sufficiently with their first-person perspective, manual responses might then be integrated with this image, leading to an erosion of the egocentric default. The tasks were not designed to adjudicate between accounts of how the content of another agent’s visual perspective might be cognitively represented. Indeed, this is one of the hardest problems in research into VPT. For example, there are mental imagery accounts recently posited as underpinning Level 2 VPT problems (Ward et al., 2019, 2020), but some scholars propose simpler, short-cut heuristics (Samuel, Eacott, et al., 2022; Samuel et al., 2021) and have argued against the plausibility of imagistic representations of others’ visual experience (Cole & Millett, 2019; Cole et al., 2020, 2022). The distinction between an embodiment account of VPT, which concerns the engagement of spatial and motor representations, and a mental imagery account, which concerns the quasi-perceptual content of the experience of a scene, is twofold. Firstly, the embodiment account *directly predicts* that motor representations are integrated with target spatial perspectives. In contrast, to our knowledge, no one has formally posited that first-person manual action should be integrated with image-like representations of others’ visual experience. Secondly, embodiment implies the transformation of one’s frame of reference *in order to* understand a perspective, whereas a mental image of another’s perspective would be more an outcome of a (unknown) process. Our data do not directly allow us to select between these two options. Nevertheless, we feel that the specific prediction about manual action made by an embodiment account would privilege this as an explanation for our findings, and currently at least there is more evidence in favour of embodied perspective taking than mental imagery accounts of VPT.

It is important to caveat interpretations of ‘real life’ social cognition from confected tasks such as those conducted here. Historically, perspective taking tasks have often employed avatars, computerised stimuli, fictional characters, photographs and so on, as is the case across a great deal of social cognition research more broadly. These are attempts to simulate perspective taking in the real world, but do not involve thinking about a real person’s experience or a typical scene. Many also apply multiple, speeded trials in a way that would be highly unnatural in real life. In sum, tasks such as those presented here do not benefit from ecological validity. In this sense, we have shown only that the egocentric advantage can be reduced under artificial conditions. Nevertheless, a benefit of pushing the boundaries of perspective taking performance in this way is that it can (and has) revealed aspects that may have otherwise remained hidden, such as embodiment (e.g. Kessler & Thomson, 2010) and line of-sight reasoning (Michelon & Zacks, 2006). An important role of future research will be to continue to caveat (or better justify) these artificial methods, *or* transition where possible to more natural scenarios as soon as experimental control and sensitive measurement can be achieved.

Given that practice disambiguating visual stimuli from other perspectives can eliminate egocentricity in manual responses, the next question is to ask to what extent practice in other domains of perspective taking might show. Thorsten Erle and colleagues have shown that making left/right judgements from another agent’s perspective led participants to approximate their guesses to trivia questions and trust the agent more (Erle et al., 2018; Erle & Topolinski, 2017). What is not clear is whether the effect of perspective taking might extend to other domains. Future research could look at whether a period of time spent considering another agent’s preferences, opinions and emotions might ‘rub off’ on the perspective taker’s own. If egocentricity is flexible even in a domain as ostensibly utilitarian as manual action, it could be that state and even trait characteristics could also be warped by perspective taking. Part of the answer to this question may lie in whether embodied perspective taking in spatial perspective taking (SPT) tasks is conceptually similar to mental simulation in some accounts of Theory of Mind (Gallese & Goldman, 1998; A. Goldman & de Vignemont, 2009; A. I. Goldman, 2006; A. I.Goldman & Jordan, 2013). That is, does the process of making spatial judgements from others’ perspectives engage similar mechanisms to the process of simulating another’s abstract belief, such as *Sally [falsely] believes that the marble is in the basket*? If so, then we should expect that frequently inferring others’ beliefs, or frequently acting upon those beliefs (e.g. physically searching in the basket), should negatively impact the integrity of our own (true) beliefs or egocentric actions upon them. To date, there is only limited and nascent evidence of this type of ‘belief infection’, in chimpanzees, whose search actions are biased by humans’ false beliefs about the location of the target object (Lurz et al., 2022; and in humans: Samuel et al., in preparation).

In conclusion, whereas egocentricity is a hindrance when taking other perspectives, it is advantageous when we need to interact physically with our environment. Our results show that even this useful egocentricity is flexible, prone to disappear and even reverse if we are given practice taking other perspectives.

## Supplemental Material

sj-csv-2-qjp-10.1177_17470218251341289 – Supplemental material for How to eliminate (and even reverse) egocentric bias in perspective taking Supplemental material, sj-csv-2-qjp-10.1177_17470218251341289 for How to eliminate (and even reverse) egocentric bias in perspective taking by Steven Samuel, Geoff G. Cole, Madeline J Eacott and Rebecca Edwardson in Quarterly Journal of Experimental Psychology

sj-csv-3-qjp-10.1177_17470218251341289 – Supplemental material for How to eliminate (and even reverse) egocentric bias in perspective taking Supplemental material, sj-csv-3-qjp-10.1177_17470218251341289 for How to eliminate (and even reverse) egocentric bias in perspective taking by Steven Samuel, Geoff G. Cole, Madeline J Eacott and Rebecca Edwardson in Quarterly Journal of Experimental Psychology

sj-csv-5-qjp-10.1177_17470218251341289 – Supplemental material for How to eliminate (and even reverse) egocentric bias in perspective taking Supplemental material, sj-csv-5-qjp-10.1177_17470218251341289 for How to eliminate (and even reverse) egocentric bias in perspective taking by Steven Samuel, Geoff G. Cole, Madeline J Eacott and Rebecca Edwardson in Quarterly Journal of Experimental Psychology

sj-csv-6-qjp-10.1177_17470218251341289 – Supplemental material for How to eliminate (and even reverse) egocentric bias in perspective taking Supplemental material, sj-csv-6-qjp-10.1177_17470218251341289 for How to eliminate (and even reverse) egocentric bias in perspective taking by Steven Samuel, Geoff G. Cole, Madeline J Eacott and Rebecca Edwardson in Quarterly Journal of Experimental Psychology

sj-docx-1-qjp-10.1177_17470218251341289 – Supplemental material for How to eliminate (and even reverse) egocentric bias in perspective taking Supplemental material, sj-docx-1-qjp-10.1177_17470218251341289 for How to eliminate (and even reverse) egocentric bias in perspective taking by Steven Samuel, Geoff G. Cole, Madeline J Eacott and Rebecca Edwardson in Quarterly Journal of Experimental Psychology

sj-xlsx-4-qjp-10.1177_17470218251341289 – Supplemental material for How to eliminate (and even reverse) egocentric bias in perspective taking Supplemental material, sj-xlsx-4-qjp-10.1177_17470218251341289 for How to eliminate (and even reverse) egocentric bias in perspective taking by Steven Samuel, Geoff G. Cole, Madeline J Eacott and Rebecca Edwardson in Quarterly Journal of Experimental Psychology

## Notes

1. The same power calculation was used for Experiments 2 and 4 below.

2. We had originally been concerned that embodied perspective taking might require that desired perspective locations be unobstructed (Capozzi et al., 2014). However, research has since found no variation in performance on a very similar task to the one presented here regardless of whether a desired perspective location was physically accessible or not (Samuel et al., 2023).

3. We reasoned that lateralising responses in this way made it more likely that participants would make a left-sided response to a stimulus on the left, and a right-sided response for a target on the right, from either their own perspective or the avatar’s, and thus strengthen the link between the body and the display. Supporting this view, pilot data with a version of this task in which responses were always required to be self-response showed that more unintended altercentric responses tended to be made with this method than when responding with one hand throughout. Note that this method also sometimes required participants to respond to targets on the avatar’s left with their right hand (and vice versa).

4. Here and in all further cases the prior used was the default (Cauchy) 0.707, for consistency.

5. The data for this *t*-test violated the assumption of normality, but this result was also confirmed by a Wilcoxon test, *W*(17) = 114, *p* = .08 (corrected alpha = .0125).

6. The same comparison using log transformed values (dealing therefore with the deviation from normality in one of the cells) produced the same result, *t*(34) = 3.109, *p* = .004 (corrected alpha = .0125), *d* = 1.04. Both these *t*-tests violated the assumption of homogeneity of variances, therefore we also conducted an unplanned Mann–Whitney test on the same data. This proved non-significant with the adjusted alpha for multiple comparisons, *U*(36) = 239, *p* = .014 (corrected alpha = .0125). For the comparison of other-response trials, there were no such issues.

7. The assumption of homogeneity of variances was not met for this comparison, including after log transforming values, but a Mann–Whitney test confirmed this result, and was statistically significant even after correcting for multiple comparisons, *U*(36) = 242, *p* = .011 (corrected alpha = .0125).

8. The vast majority of exclusions (36) occurred because that participant’s accuracy across the task as a whole was below 50%. In the remaining seven cases, accuracy was higher than 50% overall but lower than 40% on one perspective. It is difficult to interpret whether the difficulty these participants experienced concerned difficulty taking perspectives, difficulty switching frequently between them or making manual responses according to them.
