# Supplementary material for: How to eliminate (and even reverse) egocentric bias in perspective taking
Source: Q J Exp Psychol (Hove). 2025 May 8;79(2):338–54. doi: 10.1177/17470218251341289 (PMC12796011; doi:10.1177/17470218251341289)
Supplement: sj-docx-1-qjp-10.1177_17470218251341289 – Supplemental material for How to eliminate (and even reverse) egocentric bias in perspective taking [file sj-docx-1-qjp-10.1177_17470218251341289.docx]

**Instructions**

**1. Setting up**

***To begin, if you have a phone or anything else that might flash, ring, buzz or disrupt your attention please ensure that it will not for the duration of the experiment. Interruptions influence performance, in ways we cannot detect, and can change our results quite drastically. Thank you!***

The experiment will take place in this room and will take approximately 30 minutes. It involves two computer-game like activities. The first one uses this computer, the second one uses the other computer in the room. When you finish the first activity on this computer, you will be prompted to change to the other desk and follow the instructions for the second activity there.

The first activity is a visual perspective-taking game. Pay close attention to the instructions, then there will be a practice session which will tell you how you are doing.

To begin, first notice that in front of you is a button box and a computer screen. Please do not move or adjust either from their present locations.

There’s also a pair of headphones. Please put these on and keep them on until you leave this desk. You’ll need to listen to instructions during the task.

Four of the keys on the button box are covered with stickers (two yellow, two pink). One has a blue sticker. The blue button is a start button. The other buttons with stickers are the ones you need for the main activity. You’ll be reminded later, but you should use your forefinger of your left hand to press the yellow buttons, and the forefinger of your right hand to press the pink buttons.


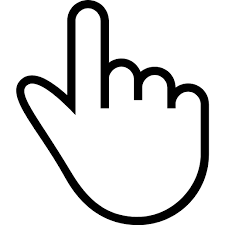

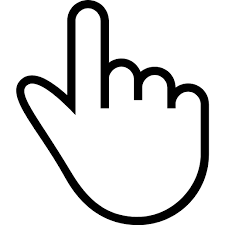


**2. The Visual Perspective-Taking Activity - Practice**

In the Visual Perspective-Taking task you will see a grid of four squares on the screen (see picture below). Two of the squares will contain digits. There will also be a woman, seen from above, wearing a red cap. She will always be on either the left or right of the grid, looking at it and the digits within it. For example, here she can see a 6 and a 9.


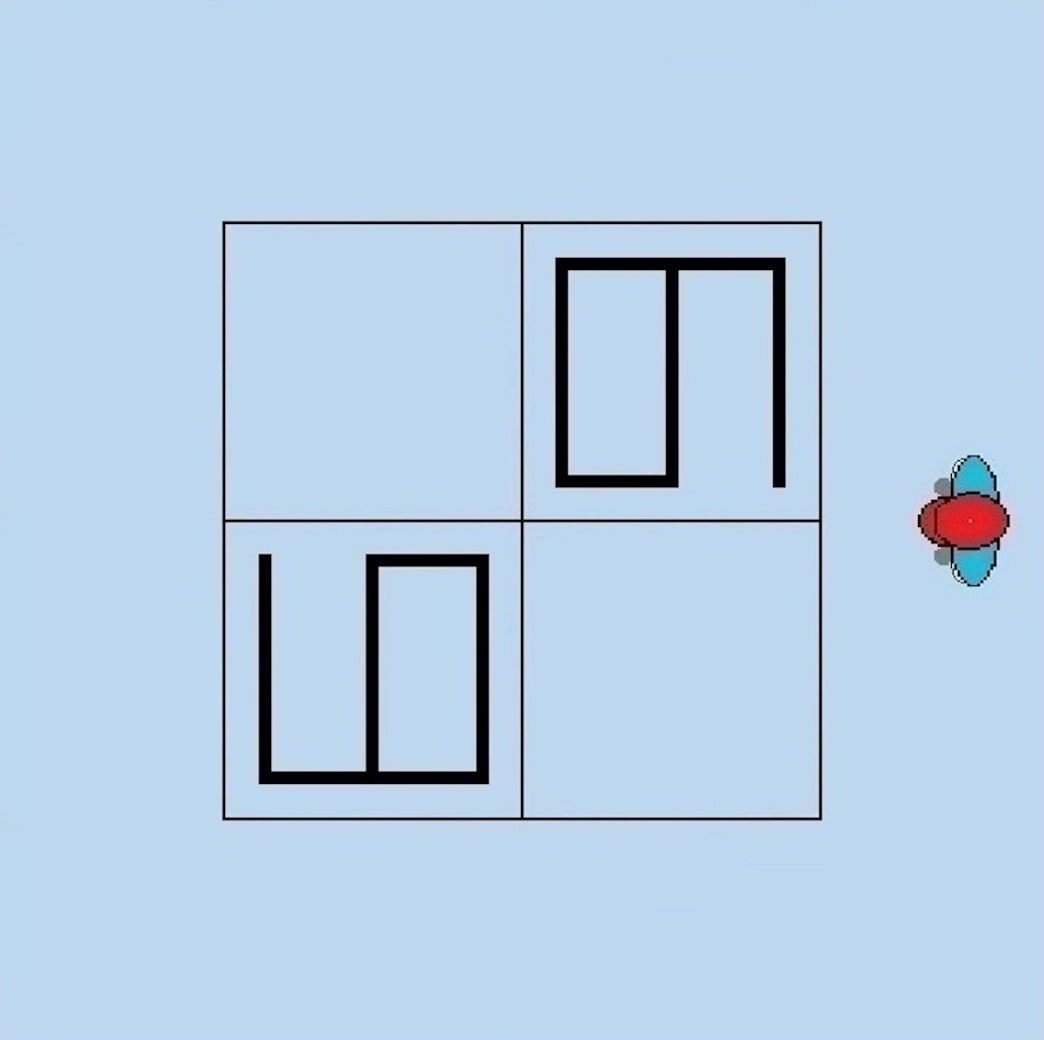


**BOTTOM LEFT**

**TOP LEFT**

**The aim of the experiment is to locate which of the two digits is the 6 from the woman’s perspective**. You then press one of the buttons on the button box to indicate where it is. **However, there are two possible answers**. For example, in the image you just saw, the 6 is in the *bottom* left corner (as you look at it). However, from her perspective, the 6 is in the *top* left corner.

Before each grid you will hear a word, either ‘You’ or ‘Her’. **When you hear ‘You’, you should press the button that indicates where the 6 is *as you see it*** (the bottom left button in the above example). **When you hear ‘Her’, you should press the button according to *where the woman sees it***, which in this case is the top left button.

To make this clearer, here’s how the same grid looks from the woman’s perspective. As you can see, the 6 is in the top left from the woman’s perspective. You’ll never actually be shown the woman’s perspective though, so you have to imagine it.


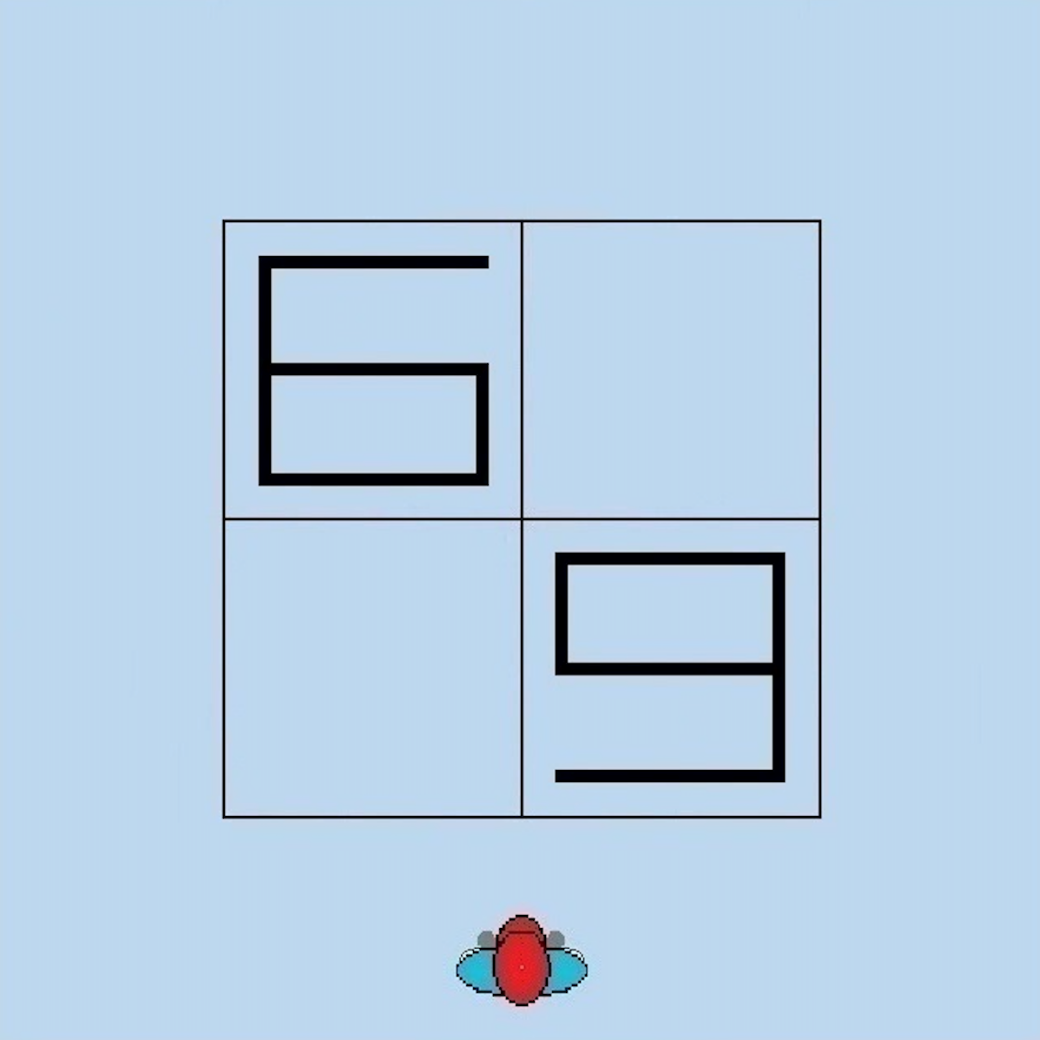


Here’s another example. The aim is always to find the 6 from the woman’s perspective, and then to press a button to say where it is as you see it (‘You’) or as she sees it (‘Her’).

***Which button should you press for ‘You’ and which button for ‘Her’? Turn over the page to check your answer.***


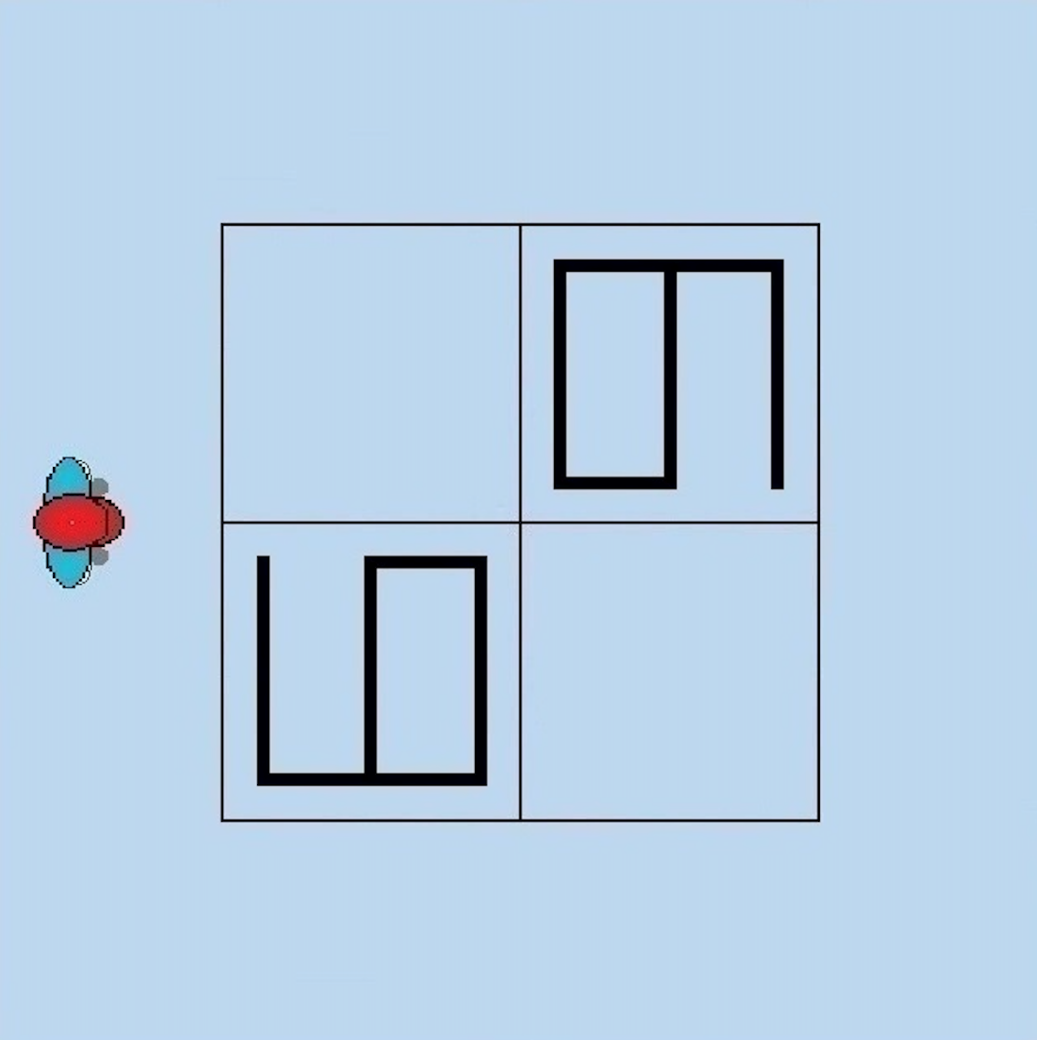


If you hear ‘You’, the correct answer is the *top right* button (see below). If you hear ‘Her’, the correct answer is the *top left* button. In fact, the woman’s view of this grid is precisely the same as her view of the previous grid.


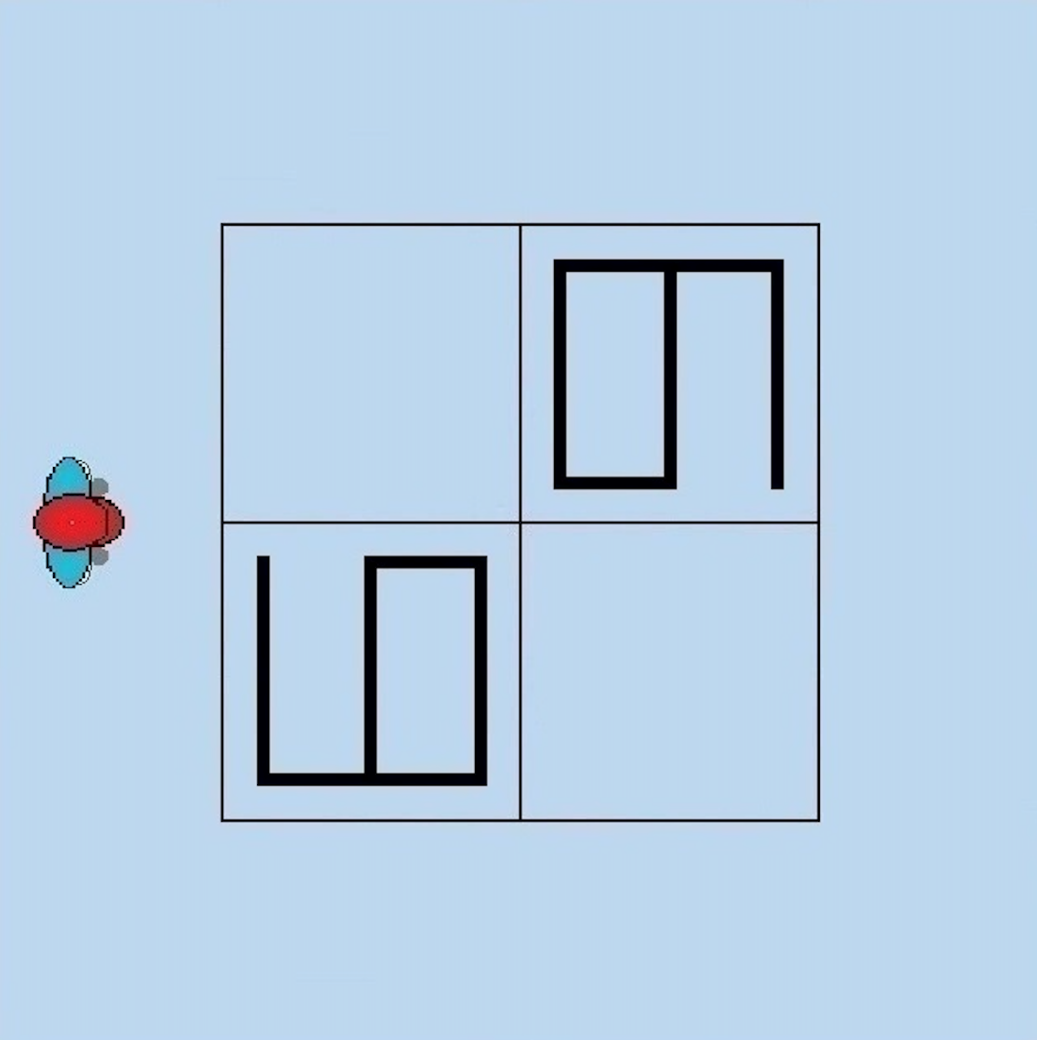


**TOP LEFT**

**TOP RIGHT**

Remember to use your left forefinger to press the two yellow keys and your right forefinger to press the two pink keys. See the arrows in the image above: a left-sided yellow key is required if the instructions is ‘Her’, but a right-sided pink key is required if the instruction is ‘You’.

If you have any questions at this stage, feel free to leave the room and ask the experimenter. If you’re ready for the practice, read on!

In the practice, you’ll hear ‘You’ or ‘Her’ before you see the grid and where the woman is standing. When you do, press the button you think is correct **as quickly and as accurately as possible**. **The screen will turn green if you are correct, or red if you are incorrect, or if you run out of time**.

Press the blue button to begin the practice. When the practice is over, read the last bit here.

**3. The Visual Perspective-Taking Activity – Main task**

If you made mistakes in the practice and you don’t know why (the screen kept going red) please speak to the experimenter after you finish the practice. Otherwise, press the blue button again to begin the activity. ***From now on, you will not be told if you are correct or not!***

Good luck!
